# TBE方案预处理自体造血干细胞移植治疗原发中枢神经系统淋巴瘤的临床分析

**DOI:** 10.3760/cma.j.cn121090-20241215-00572

**Published:** 2025-11

**Authors:** 俊丽 陈, 艺 马, 瑞青 赵, 秀斌 肖, 喜林 陈, 顺宗 袁, 世华 赵, 云 鲁, 泓浩 高, 玥琦 王, 华 殷, 娜娜 程, 盼 封, 笑然 白, 文荣 黄

**Affiliations:** 中国人民解放军总医院第五医学中心血液病医学部淋巴瘤-浆细胞疾病专科，北京 100071 Department of Lymphoma & Plasma Cell Disease, Senior Department of Hematology, the Fifth Medical Center of PLA General Hospital, Beijing 100071, China

**Keywords:** 造血干细胞移植, 原发中枢神经系统淋巴瘤, 塞替派, 白消安, 依托泊苷, Hematopoietic stem cell transplantation, Primary central nervous system lymphoma, Thiotepa, Busulfan, Etoposide

## Abstract

**目的:**

探讨TBE（塞替派+白消安+依托泊苷）方案预处理的自体造血干细胞移植（TBE auto-HSCT）治疗原发中枢神经系统淋巴瘤（PCNSL）的疗效和安全性。

**方法:**

回顾性分析2021年11月1日至2024年4月30日在中国人民解放军总医院第五医学中心血液病医学部完成TBE auto-HSCT治疗的27例PCNSL患者临床资料。

**结果:**

27例（25例初治，2例首次复发）患者中男16例、女11例，中位年龄为57（23～72）岁，>60岁患者12例（44.4％，12/27），诊断至移植的中位时间为6.9（5.0～10.0）个月。TBE auto-HSCT使缓解深度明显增加，9例移植前部分缓解患者在移植后获得完全缓解（CR），患者总体CR率由auto-HSCT前的63.0％增加至96.3％（*P*＝0.005）。中位随访时间24.5（2.0～36.0）个月，2年无进展生存率及总生存率分别为（87.2±6.9）％和（88.6±6.2）％。最常见的3级非血液学不良事件为腹泻（18.5％，5/27），其次为细菌感染（14.8％，4/27）。1例64岁患者因系统性耐碳青霉烯肠杆菌（CRE）感染于移植后2个月死亡，移植后100 d的治疗相关死亡率为3.7％（1/27）。

**结论:**

TBE方案预处理的高剂量化疗序贯auto-HSCT治疗PCNSL可获得良好疗效与生存，治疗相关不良反应可控，老年患者可耐受。

## Abstract (en)

**Objective:**

To assess the safety and efficacy of thiotepa, busulfan, and etoposide（TBE）conditioning followed by autologous hematopoietic stem-cell transplantation（TBE auto-HSCT）in primary central nervous system lymphoma（PCNSL）patients.

**Methods:**

Clinical data from 27 PCNSL patients who received TBE auto-HSCT at the Fifth Medical Center of PLA General Hospital between November 1, 2021, and April 30, 2024, were retrospectively analyzed.

**Results:**

Twenty-seven patients［16 males, 11 females; median age 57（23–72）years］were included, with 12（44.4％, 12/27）over 60. Twenty-five had newly diagnosed PCNSL and 2 were relapsed. Median time from diagnosis to transplantation was 6.9（5.0–10.0）months. TBE auto-HSCT increased complete remission（CR）rate from 63.0 to 96.3％（*P*＝0.005）, and 9 of 10 patients in partial remission achieving CR post-transplant. Median follow-up was 24.5 months（range 2.0–36.0）. Two-year progress-free and OS rates were（87.2±6.9）％ and（88.6±6.2）％, respectively. Common grade 3 nonhematologic adverse events were diarrhea（18.5％, 5/27）and bacterial infections（14.8％, 4/27）. One patient（64 years old）died from carbapenem-resistant Enterobacteriaceae infection within 2 months post-transplant, yielding a 100-day treatment-related mortality of 3.7％（1/27）.

**Conclusion:**

TBE-conditioned high-dose chemotherapy with auto-HSCT is effective, safe, and well-tolerated in PCNSL patients, including the elderly.

原发中枢神经系统淋巴瘤（PCNSL）是一类罕见的结外非霍奇金淋巴瘤，主要累及脑组织、软脑膜、脊髓或眼球后组织且无脑外组织侵犯，90％～95％的病理类型为弥漫大B细胞淋巴瘤（DLBCL），世界卫生组织（WHO）将其归类为“原发性免疫豁免部位的大B细胞淋巴瘤”，为预后最差的淋巴瘤类型[1]。目前推荐的PCNSL治疗策略包括以高剂量甲氨蝶呤（HD-MTX）为基础的诱导化疗及后续巩固治疗，巩固治疗措施包括高剂量化疗序贯自体造血干细胞移植（high-dose chemotherapy with autologous hematopoietic stem-cell transplantation，HDC auto-HSCT）及全脑放疗（WBRT）。国际多中心的前瞻性研究证实HDC auto-HSCT疗效优于WBRT[2]–[3]，且WBRT的迟发性神经毒性发生率高，严重影响患者生活质量[4]，因此国内外指南均推荐适合移植的患者优选HDC auto-HSCT[1],[5]。含塞替派预处理方案是PCNSL患者auto-HSCT首选方案，常用TBC（塞替派+白消安+环磷酰胺）和TT/BCNU（塞替派+卡莫司汀）方案，TBC方案疗效优于TT/BCNU方案，但其治疗相关不良反应较大[6]–[7]，因此平衡HDC auto-HSCT的疗效与安全性是临床亟待解决的问题。我中心对TBC方案进行改良，以依托泊苷（VP-16）替代环磷酰胺，调整为TBE（塞替派+白消安+ VP-16）方案，本研究回顾性分析了我中心应用TBE方案预处理的auto-HSCT（TBE auto-HSCT）治疗PCNSL的疗效及安全性。

## 病例与方法

一、病例

回顾性分析2021年11月1日至2024年4月30日期间于中国人民解放军总医院第五医学中心血液病医学部淋巴瘤-浆细胞疾病专科接受TBE auto-HSCT的27例PCNSL患者的临床资料。颅脑增强磁共振成像作为治疗前评估，全身影像学检查排除中枢神经系统及眼之外部位淋巴瘤累及，常规行眼科检查及腰椎穿刺获取脑脊液化验（行脑脊液常规、生化、细胞学以及流式细胞术分析）。PCNSL诊断标准参照WHO造血与淋巴细胞肿瘤分类2022年第5版[8]，预后分层参照国际结外淋巴瘤工作组（IELSG）预后模型[9]。纳入标准：①年龄18～75岁；②所有患者均基于病理形态和免疫组织化学染色确诊为DLBCL；③美国东部肿瘤协作组评分≤3分；④移植前疗效达到部分缓解（PR）或完全缓解（CR）。排除标准：①有未控制的感染及合并严重器官功能衰竭等；②精神障碍或无法配合治疗者；③既往有其他恶性肿瘤病史。本研究通过中国人民解放军总医院医学伦理委员会审批（批件号：KY-2025-1-10-1）。

二、治疗

1. 诱导治疗：初治及复发时间距末次治疗大于12个月的患者采用一线治疗方案：利妥昔单抗（375 mg/m^2^，第0天）+HD-MTX（3.5 g/m^2^，第1天）+布鲁顿酪氨酸激酶抑制剂（奥布替尼，150 mg/d，第1～21天）。进展或难治或复发时间距末次治疗小于12个月的患者采用二线治疗方案：利妥昔单抗（375 mg/m^2^，第0天）+大剂量阿糖胞苷（HD-Ara-C）（2 g/m^2^，每12 h 1次，第1～2天）+替莫唑胺（150 mg/m^2^，第1～5天）±塞替派（30 mg/m^2^，第3天）。21 d为1个周期，诱导治疗后疗效≥PR的患者接受auto-HSCT。

2. 自体造血干细胞动员及采集：常规行原化疗方案+VP-16+重组人粒细胞集落刺激因子（rhG-CSF）（10 µg·kg^−1^·d^−1^）方案动员造血干细胞。VP-16剂量：接受HD-MTX化疗患者VP-16为1 g/m^2^×1 d；接受HD-Ara-C化疗患者VP-16为0.6 g/m^2^×1 d。采集目标：CD34^+^细胞数≥2×10^6^/kg。

3. TBE auto-HSCT：TBE方案预处理：塞替派450 mg·m^−2^·d^−1^静脉滴注，移植前第7天（−7 d）；白消安3.2 mg·kg^−1^·d^−1^静脉滴注，−6 d至−4 d；依托泊苷2 g/m^2^持续静脉输注12 h，−3 d；自体造血干细胞回输，0 d。预处理化疗期间口服苯妥英钠预防药物性癫痫发作。中性粒细胞植入定义为中性粒细胞绝对计数>0.5×10^9^/L连续3 d；血小板植入定义为血小板计数>20×10^9^/L连续7 d且脱离血小板输注。造血重建延迟定义为造血干细胞回输后中性粒细胞或血小板植入时间超过28 d。

三、疗效评价及安全性评估标准

疗效评价标准参照国际原发中枢神经系统淋巴瘤合作组（IPCG）标准[10]，分为CR、PR、疾病稳定（SD）、疾病进展（PD），总有效率（ORR）定义为获得PR及以上疗效患者比例。安全性评估标准依照美国常见不良反应术语评定标准5.0版分级标准评定。

四、随访

通过查阅住院病历、门诊病历及电话随访，随访截止日期为2024年12月15日，中位随访时间为24.5（2.0～36.0）个月。移植后100 d内与原发疾病无关的死亡定义为移植相关死亡。无进展生存（PFS）期定义为自体造血干细胞回输日至任何原因导致疾病进展、首次复发或末次随访的时间。总生存（OS）期定义为自体造血干细胞回输日至任何原因导致死亡或末次随访的时间。

五、统计学处理

应用Graphpad prism 9.5统计学软件进行数据分析。计数资料以例数（％）表示，率的比较采用*χ*^2^检验，符合正态分布的计量资料以*x*±*s*表示，组间比较采用*t*检验，不符合正态分布的计量资料用中位数（范围）表示，组间比较采用非参数检验。Kaplan-Meier法进行生存分析，并行Log-rank检验，*P*<0.05为差异具有统计学意义。

## 结果

一、基线资料

本研究共纳入27例（25例初治、2例首次复发）PCNSL患者，男女比例为16∶11，中位年龄57（23～72）岁，>60岁患者12例（44.4％，12/27），诊断至移植的中位时间为6.9（5.0～10.0）个月。27例患者病理诊断均为DLBCL，其中非生发中心起源14例（51.8％，14/27）。IELSG高危9例（33.3％，9/27），美国东部肿瘤协作组评分2～3分6例（22.2％，6/27）。9例行肿瘤组织二代基因测序，其中MYD88^L265P^突变7例、CD79B突变5例、TP53突变2例。25例初治患者采用R-MO（利妥昔单抗+MTX+奥布替尼）方案诱导治疗，2例复发患者分别采用R-TAT（利妥昔单抗+塞替派+阿糖胞苷+替莫唑胺）和R-AT（利妥昔单抗+阿糖胞苷+替莫唑胺）方案诱导治疗，诱导治疗中位周期数为5（4～6）个，诱导治疗后ORR为100％，CR率和PR率分别为63.0％（17/27）和37.0％（10/27）。

二、auto-HSCT与造血重建

27例患者单个核细胞（MNC）中位回输量为12.3（7.6～34.3）×10^8^/kg，CD34^+^细胞中位回输量为12.4（3.9～25.1）×10^6^/kg，移植期间血小板的中位输注量为2（1～10）单位。所有患者均造血重建成功，中性粒细胞及血小板中位植入时间分别为9.0（8.0～11.0）d和8.5（5.0～16.0）d。

三、移植相关不良事件

27例患者移植相关不良事件见表1，所有患者的中性粒细胞及血小板减少均为4级，3级贫血发生率为14.8％（4/27）。3级非血液学不良事件中发生率最高的是腹泻（18.5％，5/27），其次是细菌感染（14.8％，4/27）。细菌感染包括上呼吸道感染、肺部感染、肠道感染、尿路感染，1例64岁患者因系统性耐碳青霉烯类肠杆菌（CRE）感染于移植后2个月死亡。1例患者发生心房颤动伴快速心室率，未发生任何级别肾脏、神经系统不良事件，移植相关死亡率（TRM）为3.7％（1/27）。年龄≤60岁和>60岁患者的移植相关不良事件发生率差异均无统计学意义（均*P*>0.05）。

**表1:** 27例接受TBE方案预处理的自体造血干细胞移植治疗的原发中枢神经系统淋巴瘤患者移植相关不良事件分布［例（％）］

不良事件	1级	2级	≥3级	合计
贫血	8（29.6）	7（25.9）	4（14.8）	19（70.3）
粒缺伴不明原因发热	5（18.5）	6（22.2）	3（11.1）	14（51.8）
细菌感染	1（3.7）	3（11.1）	5（18.5）	9（33.3）
口腔黏膜炎	5（18.5）	4（14.8）	3（11.1）	12（44.4）
恶心	5（18.5）	13（48.1）	3（11.1）	21（77.7）
腹泻	3（11.1）	4（14.8）	5（18.5）	12（44.4）
胃肠道黏膜炎	2（7.4）	0（0）	2（7.4）	4（14.8）
消化道出血	0（0）	0（0）	2（7.4）	2（7.4）
谷丙转氨酶升高	8（29.6）	3（11.1）	2（7.4）	13（48.1）
心房颤动	0（0）	1（3.7）	0（0）	1（3.7）
B型钠酸肽升高	3（11.1）	0（0）	0（0）	3（11.1）

**注** TBE：塞替派+白消安+依托泊苷；粒缺：中性粒细胞缺乏

四、疗效与生存

所有患者均于移植后1个月完成疗效评价。移植后CR率为96.3％，较移植前明显提升［96.3％（26/27）对63.0％（17/27），*P*＝0.005］，9例（90％，9/10）移植前PR患者移植后获CR，2例首次复发的患者移植后均获CR。>60岁患者［83.3％（10/12）对58.3％（7/12），*P*＝0.371］、IELSG高危患者［88.9％（8/9）对55.6％（5/9），*P*＝0.294］以及MYD88^L265P^突变患者［100％（7/7）对57.1％（4/7），*P*＝0.192］移植后CR率均提升，尽管差异无统计学意义。

中位随访24.5（2.0～36.0）个月，患者总体中位PFS及OS期均未达到，2年PFS率及OS率分别为（87.2±6.9）％和（88.6±6.2）％。>60岁患者OS差于≤60岁患者［2年OS率：（75±12.5）％对100％，*P*＝0.048］；而PFS差异无统计学意义［2年PFS率：（81.8±11.6）％对（90.9±8.7）％，*P*＝0.354］（图1）。随访期间3例患者（均为非生发中心起源）进展，其中2例（1例为EB病毒阳性伴TP53突变）老年初治IELSG高危男患者分别于移植后第3个月、第6个月进展，余1例初治IELSG中危患者于移植后第14个月进展。共3例患者死亡，年龄分别为64岁、62岁、65岁，其中1例因CRE感染死亡，2例因疾病进展死亡，非复发死亡率（NRM）为3.7％。

**图1:**
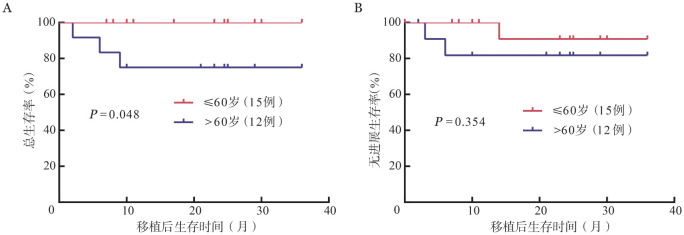
≤60岁与>60岁组原发中枢神经系统淋巴瘤患者经TBE（塞替派+白消安+依托泊苷）方案预处理的自体造血干细胞移植后总生存（A）和无进展生存（B）曲线

## 讨论

PCNSL是化放疗高度敏感的肿瘤，但化疗药物须在脑脊液和中枢神经系统实质中达到足够的浓度才能发挥药效，因此PCNSL用药标准为药物高浓度以及中枢神经系统的高生物利用度。鉴于塞替派较高的中枢神经系统渗透率［（脑脊液药物浓度/血浆药物浓度）×100％］，以塞替派为基础的预处理方案是HDC auto-HSCT的首选方案，其中TBC方案具有明显的PFS及OS优势，但治疗相关不良反应不容忽视[7]。该方案中，塞替派与白消安的中枢神经系统渗透率均>80％[11]，而环磷酰胺则≤15％[12]，联合环磷酰胺能否使PCNSL患者从中获益尚未知，且其可能附加药物不良反应。Postmus等[13]研究发现，静脉输注高剂量VP-16（0.9～2.5 g/m^2^）可使脑脊液药物浓度达到0.54 µg/ml，且持续静脉输注的用药方式可维持长时间药物浓度暴露，利于脑组织穿透[14]，VP-16在复发难治中枢神经系统淋巴瘤以及儿童急性淋巴细胞白血病累及中枢神经系统的临床研究中亦显示出高效性[15]–[16]，而高剂量VP-16的剂量限制性不良反应主要为骨髓抑制，其他不良反应均可控[11]。基于以上研究结果并结合VP-16药代动力学（VP-16静脉输注后50 h，血浆药物浓度几乎为0[17]），我中心采用VP-16（2.0 g/m^2^持续静脉输注12 h，−3d）替代环磷酰胺的TBE autoHSCT作为PCNSL巩固治疗手段，获得了良好的肿瘤控制率及生存，同时不良反应可控，移植相关死亡率低。

一项美国的Meta分析汇总2003年至2017年期间43项关于PCNSL的HDC auto-HSCT临床研究[7]，结果显示：TBC autoHSCT巩固治疗后ORR达90％，2年、5年的PFS率分别为86％、81％，OS率则分别为90％、81％，显著优于TT/BCNU（64％、46％；75％、70％）以及BEAM方案（43％、无法获得；71％、无法获得）。本研究中>60岁患者占比为44％（12/27），而诱导治疗及TBE autoHSCT巩固治疗后ORR均为100％，移植后CR率提高了52.9％，缓解深度的增加转化为生存获益，2年PFS率及OS率分别为（87.2±6.9）％和（88.6±6.2）％，本研究结果证实TBE方案预处理的HDC auto-HSCT同样可获得良好的疾病缓解与生存，并不劣于经典的TBC方案。

HDC auto-HSCT不同的预处理方案导致了生存差异，而治疗相关不良反应可能影响预处理方案的选择甚至患者预后。来自纪念斯隆-凯特琳癌症中心关于TBC auto-HSCT治疗中枢神经系统淋巴瘤的研究结果显示：TBC方案主要不良事件为3/4级黏膜炎，发生率为81％，3级中性粒细胞缺乏伴发热和感染（包括细菌和真菌感染）发生率分别为95％、44％，3级腹泻和心血管事件发生率则分别为28％、26％，TRM为7％[18]。而加利福尼亚大学洛杉矶分校的一项关于TBC auto-HSCT治疗中枢神经系统淋巴瘤的回顾性分析显示：89.6％患者发生黏膜炎，3级及以上黏膜炎发生率为35.4％；细菌感染发生率为58.3％；肾脏损害及环磷酰胺相关出血性膀胱炎发生率分别为16.7％和8.3％；心脏毒性不良事件发生率为16.7％，其中2.1％可疑是环磷酰胺相关心肌病；TRM为8.3％，死亡原因为重症感染[19]。因此采用TBC方案预处理的HDC auto-HSCT应尽量在有经验的移植中心进行且移植患者需严格筛选。而TBE方案不良反应可控，3级黏膜炎（口腔和胃肠道）、腹泻及细菌感染发生率较低。黏膜炎发生率和发生级别的降低有利于感染防控，本研究中仅1例患者因CRE感染而死亡，未出现其他重症感染。心血管事件为1例心房颤动伴快速心室率，未发生肾及神经系统不良事件，TRM和NRM均为3.7％，低于上述TBC auto-HSCT相关研究结果，提示我中心TBE方案具有更高的安全性。

影响PCNSL预后的因素除巩固治疗方案的疗效与不良反应外，还包括一些宿主因素，例如年龄、体能状况、涉及肿瘤发病机制的遗传学因素等。法国眼-脑淋巴瘤网络数据库的5年随访结果显示：年龄>60岁、男性、Karnofsky评分<70分以及较差的诱导疗效，均与较短的OS期相关[20]。本研究中3例死亡患者均为老年男性，2例因本病死亡，1例因感染CRE死亡，生存分析显示>60岁患者OS差于≤60岁患者（*P*＝0.048），与以上研究结果一致，但仍需增加样本量及延长随访时间进一步验证。有学者认为DLBCL非生发中心起源及TP53突变与PCNSL较差预后相关[21]–[22]，MYD88^L265P^突变是诊断和靶向治疗的分子生物学标志，亦提示预后不良[23]。本研究中3例复发患者均为非生发中心起源，1例死亡患者伴TP53突变，而7例MYD88^L265P^突变患者随访期间疗效持续CR，TBE auto-HSCT能否改善或克服MYD88^L265P^突变患者的不良预后仍需进一步观察。

综上所述，本研究TBE方案预处理的HDC auto-HSCT的疗效与生存不劣于经典TBC方案，且治疗相关不良反应减少，后续仍需增加样本量、延长随访时间进一步观察TBE auto-HSCT在真实世界的远期生存与安全性，使更多PCNSL患者从中获益。
